# Fibrin Glue-aided, Instant Epicardial Placement Enhances the Efficacy of Mesenchymal Stromal Cell-Based Therapy for Heart Failure

**DOI:** 10.1038/s41598-018-27881-5

**Published:** 2018-06-21

**Authors:** Kazuya Kobayashi, Yuki Ichihara, Nobuko Tano, Laura Fields, Nilaani Murugesu, Tomoya Ito, Chiho Ikebe, Fiona Lewis, Kenta Yashiro, Yasunori Shintani, Rakesh Uppal, Ken Suzuki

**Affiliations:** William Harvey Research Institute, Barts and The London School of Medicine and Dentistry, Queen Mary University of London, London, United Kingdom

**Keywords:** Stem-cell research, Preclinical research, Translational research

## Abstract

Transplantation of mesenchymal stromal cells (MSCs) is a promising new therapy for heart failure. However, the current cell delivery routes result in poor donor cell engraftment. We therefore explored the role of fibrin glue (FG)-aided, instant epicardial placement to enhance the efficacy of MSC-based therapy in a rat ischemic cardiomyopathy model. We identified a feasible and reproducible method to instantly produce a FG-MSC complex directly on the heart surface. This complex exhibited prompt, firm adhesion to the heart, markedly improving initial retention of donor MSCs compared to intramyocardial injection. In addition, maintenance of retained MSCs was enhanced using this method, together contributing the increased donor cell presence. Such increased donor cell quantity using the FG-aided technique led to further improved cardiac function in association with augmented histological myocardial repair, which correlated with upregulation of tissue repair-related genes. We identified that the epicardial layer was eliminated shortly after FG-aided epicardial placement of MSCs, facilitating permeation of the donor MSC’s secretome into the myocardium enabling myocardial repair. These data indicate that FG-aided, on-site, instant epicardial placement enhances MSC engraftment, promoting the efficacy of MSC-based therapy for heart failure. Further development of this accessible, advanced MSC-therapy is justified.

## Introduction

Transplantation of mesenchymal stromal cells (MSCs) is an emerging approach for the treatment of diseases, which at present lack effective therapies, including heart failure^1^. Although cardiomyogenic potency of transplanted MSCs may not be sufficient to improve cardiac function *in vivo*, these cells are able to secrete specific growth factors, cytokines, chemokines, microRNAs, and/or exosomes, which are potent to enhance repair, recovery and regeneration of the damaged myocardium (termed “paracrine effect”)^1–3^. In addition, this cell type may be used for allogeneic cell transplantation without an immunosuppressive reagent presumably due to their immunomodulation properties^1,4^. It could represent a substantial advantage of MSCs as a donor for cell-based therapy, although this feature remains disputable and needs further elucidation^5^. However, in order to establish MSC-based therapy as a widely-adopted treatment for heart failure, current cell-delivery methods need to be further optimised^6^. The common cell-delivery routes include direct intramyocardial (IM) injection and intracoronary injection. Either method is associated with poor retention and survival of donor cells, and thereby result in inadequate donor cell engraftment in the heart, limiting the potential of this treatment. Furthermore, IM injection generates isolated clusters of donor cells, which is associated with acute inflammation. This potentially induces ventricular arrhythmias due to disruption of electrical propagation within the myocardium and re-entry formation^7,8^. On the other hand, intracoronary injection carries the risk of coronary embolism, which is particularly critical when infusing MSCs into a diseased and narrowed coronary artery^6,9,10^.

Epicardial placement is an alternative route for MSC-delivery to the heart^6,9^. This is localized placement (not injection) of donor cells onto the epicardial heart surface. We have demonstrated that this delivery method, in the form of scaffold-free cell-sheets, increased engraftment of donor MSCs in the heart as compared to IM injection in rat models of acute myocardial infarction (MI) and ischemic cardiomyopathy (ICM)^2,3^. The increased engraftment of donor MSCs resulted in amplified myocardial repair and augmented cardiac function recovery. However, standardized production of homogeneous cell-sheets as well as their transport and handling are technically challenging, constraining widespread clinical establishment of this method. We therefore explored a more accessible technique to achieve effective epicardial placement of MSCs.

Fibrin glues (FG) are a widely-used medical product for hemostasis and wound healing^11^. Within seconds of mixing concentrated fibrinogen and thrombin solutions, thrombin enzymatically cleaves fibrinogen and forms fibrin polymers, presented as a hydrogel that is able to firmly adhere to the tissue. As FG can serve as a suitable three-dimensional scaffold for cultured cells, this biomaterial has been widely used as a cell culture system, a component of tissue engineering constructs or as an injectable material mixed with cells^12^. The capabilities of this biomaterial to enhance therapeutic effects of cell transplantation therapy have been increasingly recognized in various diseases in bone, cartilage, cornea, blood vessel, tendon and heart^12^. This includes IM injection of an FG-MSC mixture^13,14^ and patches of the prefabricated FG-MSC constructs^15,16^. However, IM injection of FG is associated with a high risk of thromboembolism^17^, while the prefabricated FG-MSC construct again presents a more complex therapeutic solution in terms of handling, logistics and quality control. We believe that instant production of the FG-MSC complex directly on the heart surface is safe, easy to apply, and may draw the maximum potential of this approach. By delivering ready-to-use, quality-controlled MSCs together with the safety-proven fibrinogen and thrombin solutions, logistics and quality control of this product is straightforward, without the requirement for a cell processing facility in each hospital. Thus, this user-friendly technique has a great potential to become a widely adopted MSC-based therapy for heart failure. As such, this study aimed to establish feasibility, safety and efficacy of FG-aided, instant epicardial placement of MSCs in a rat heart failure model. We also aimed to uncover the mechanistic factors underpinning the role of FG to achieve effective epicardial placement of MSCs for the treatment of heart failure.

## Results

### FG-aided, instant epicardial placement markedly improved both initial retention and survival of donor MSCs compared to IM injection

**Figure 1:**
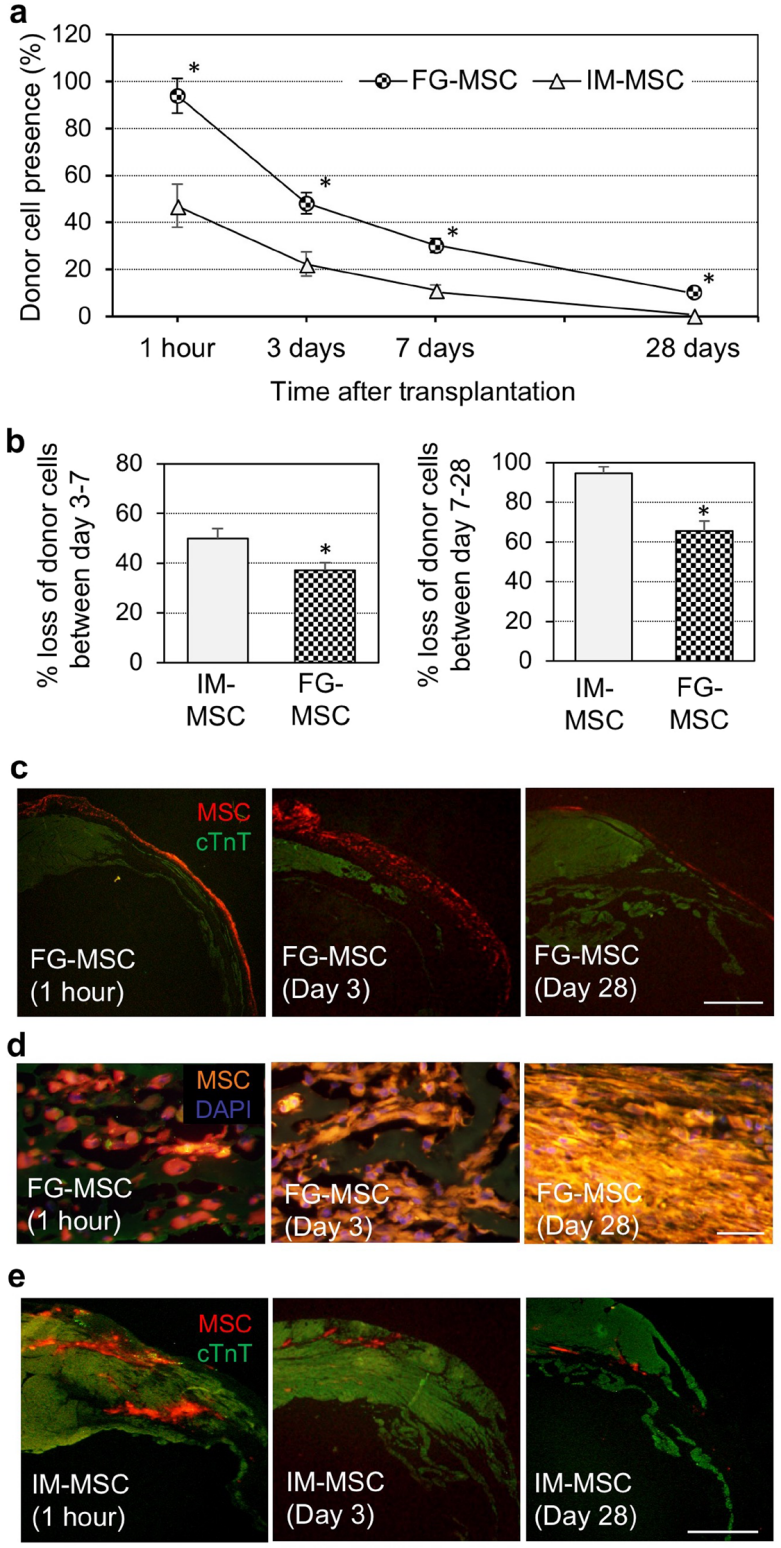
Improved initial retention and survival of MSCs transplanted using FG-aided, instant epicardial placement. (**a**) Four weeks after coronary artery ligation, female rats were transplanted with male MSCs using FG-aided, instant epicardial placement (FG-MSC group) or intramyocardial injection (IM-MSC group). Quantitative PCR for the male specific *Sry* gene detected increased retention of donor cells (shown as % of the total cell number transplanted) in the FG-MSC group compared to the IM-MSC group. ^†^ *p* < 0.05 *vs*. the corresponding time point value of the IM-MSC group, n = 5 or 6 in each point. (**b**) % loss of retained MSCs between day 3 and 7 (left panel) and between day 7 and 28 (right panel) were calculated from the data shown in Fig. 1a. (**c**) Immunofluorescence analysis demonstrated that transplanted MSCs (CM-DiI-labeled) in the FG-MSC group persistently retained at the surface of the heart. Representative images from 5 or 6 hearts at each time point are presented. Scale bars = 1 mm. cTnT, cardiac troponin T. (**d**) High-magnification observations of the FG-MSC complex demonstrated gradual elimination of FG, which was detectable with the green auto-fluorescence between CM-DiI-labeled (orange) MSCs, over time. Scale bars = 50 µm. (**e**) Immunofluorescence showed that donor cells (CM-DiI-labeled; orange) in the IM group formed cell-clusters within the myocardium, which decreased in size over time. Scale bars = 1 mm.

Female Lewis rats with post-MI ICM were transplanted with 4 × 10^6^ MSCs, isolated from bone marrow of syngeneic male Lewis rats, using either FG-aided instant epicardial placement (FG-MSC group) or IM injection of MSC suspension (IM-MSC group). For epicardial placement, 40 μl of fibrinogen/MSC suspension and 40 μl of thrombin solution was simultaneously added on to the heart surface in a dropwise manner. This resulted in prompt generation of FG incorporating MSCs, which covered the infarct and peri-infarct myocardial areas as we targeted. This complex quickly and firmly adhered to the heart surface and remained *in situ* without dripping off. Real time PCR analysis of the male specific *Sry* gene demonstrated that the initial (1 hour after transplantation) retention of donor cells was markedly increased in the FG-MSC group compared to the IM-MSC group (Fig. 1a). Thereafter, donor cell presence was reduced in both groups; however, throughout the time course up to day 28, greater donor MSC presence was consistently observed in the FG-MSC group. Of note, the day 3 donor cell presence in the FG-MSC group was equivalent to 1-hour MSC presence in the IM-MSC group. Furthermore, calculated donor cell loss ratios between day 3 and 7 post-transplantation and between day 7 and 28, were both attenuated in the FG-MSC group (Fig. 1b), suggesting enhanced maintenance of retained MSCs after FG-aided epicardial placement.

### MSCs engrafted using FG-aided epicardial placement remained on the heart surface

**Figure 2:**
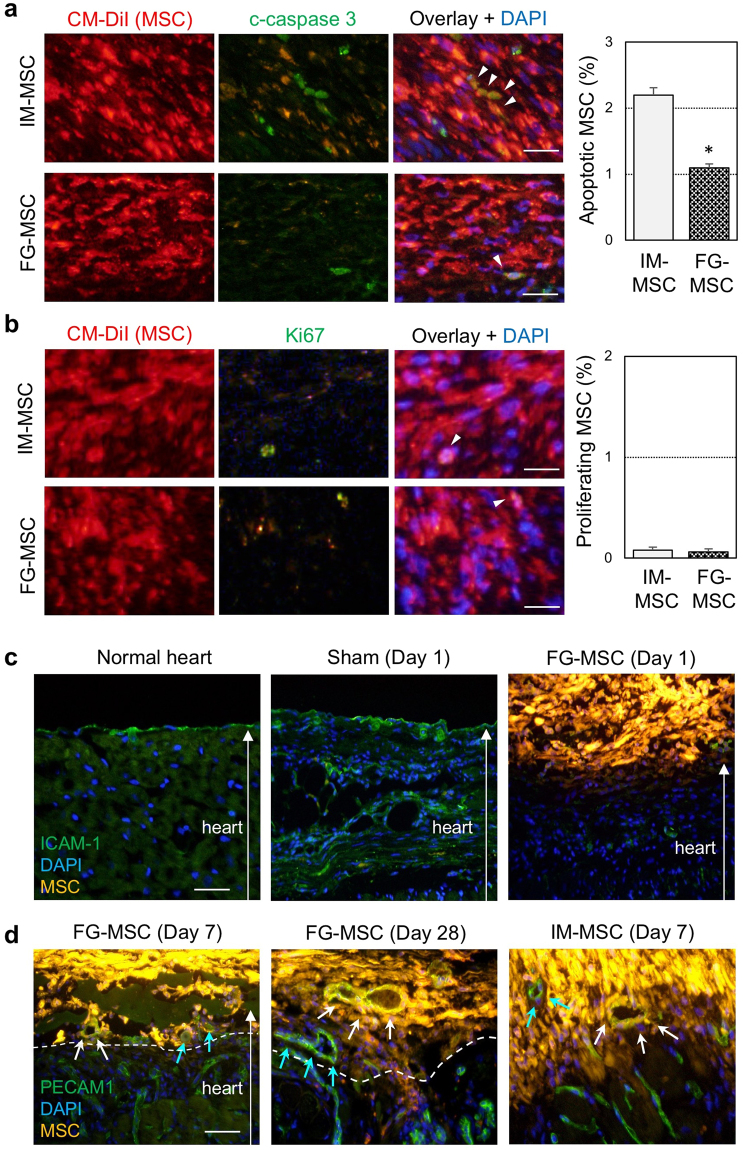
Elimination of the epicardium after FG-aided epicardial placement of MSCs. (**a**) Immunohistostaining for cleaved caspase 3 (c-caspase 3) showed a reduction of apoptosis of donor MSCs (white arrowheads) in the FG-MSC group compared to the IM-MSC group at day 3 after cell therapy. Orange signals in the c-caspase 3 panels were leaked fluorescence from the highly bright CM-DiI (red). Scale bars = 50 µm. *p < 0.05, n = 6 in each group. (**b**) Immunostaining for Ki67 showed rare proliferation of donor MSCs (white arrowheads) at day 3 after cell therapy in either group. Orange signals in the Ki67 panels were leaked fluorescent signals from the bright CM-DiI (red). Scale bars = 40 µm. n = 6 in each group. (**c**) Immunohistostaining analysis detected an ICAM-1^+^ monolayer of the epicardium in the normal (no-MI) heart and the heart of the Sham group (sham treatment in ICM rats). This epicardium disappeared by day 1 after FG-aided, instant epicardial placement of MSCs (labeled with CM-DiI; yellow). CM-DiI is originally red/orange, but CM-DiI-labeled MSCs exhibit yellow color in the figure. This is because CM-DiI-labeling of MSCs were extremely intense, and the strong red fluorescence leaked into the green channel. This was particularly the case when the labelled MSCs densely aggregated. Scale bar = 100 µm. (**d**) Immunohistostaining analysis detected PECAM1^+^ (green) and CM-DiI^+^ (yellow) vessels, suggesting trans-differentiation of donor MSCs to endothelial cells in both FG-MSC and IM-MSC groups (white arrows). As explained above, CM-DiI-labeled MSCs exhibited yellow color, but these yellow cells were distinguishable from the PECAM1^+^CM-DiI^+^ cells, which showed a different yellow color. In addition, PECAM1^+^ (green) but CM-DiI^−^ vessels were observed, indicating host-derived endothelial cells (blue arrows). Scale bar = 100 µm.

Immunohistostaining revealed that the majority of donor MSCs in the FG-MSC group were retained on the epicardial surface until day 28 (Fig. 1c). At 1 hour after placement, the FG-MSC complex covered all targeted areas of the infarct and surrounding border areas evenly. The FG-MSC layers were generally compacted at 1 hour, but became more spread by day 3 as donor MSCs became more elongated (Fig. 1d). However, this layer decreased in size by day 28. One hour after epicardial placement, FG was observed surrounding, round-shaped donor MSCs within the FG-MSC complex (Fig. 1d). At day 3, FG was still observed occasionally between MSCs, which showed an elongated morphology. At day 28, MSCs were more densely accumulated, and FG was hardly observed, suggesting degradation and/or absorption of FG by this time point after epicardial placement. In contrast, transplanted MSCs in the IM-MSC group formed isolated cell-clusters in the myocardium, while the size of these clusters decreased gradually over time (Fig. 1e). Apoptotic death of transplanted MSCs, assessed with cleaved caspase 3 staining, was reduced in the FG-MSC group compared to the IM-MSC group (Fig. 2a). On the other hand, proliferation of donor MSCs, as measured using immunostaining for Ki67, was not different between the groups; Ki67^+^ MSCs were rarely detected in either IM-MSC and FG-MSC group (Fig. 2b). We did not observe any cardiac Troponin T^+^ cardiomyocytes derived from CM-DiI-labelled donor MSCs indicating a lack of cardiomyogenic differentiation of donor MSCs. In addition, there were no Oil-red O^+^ adipocytes or Alizarin red^+^ osteocytes originated from DiI^+^ donor MSCs at day 28 (Supplemental Fig. S1), indicating safety of this treatment.

### The epicardial cell layer was eliminated after FG-aided placement of MSCs

It is generally agreed that transplanted MSCs improve cardiac function *via* the paracrine effect based on their secretome^18^. Given that the majority of donor MSCs were retained on the heart surface after FG-aided epicardial placement (Fig. 1), we cautiously considered that the presence of the epicardial cell layer might pose a barrier to epicardially-placed MSCs in terms of communicating with the host myocardium to establish a paracrine effect. In this regard, we found that the ICAM-1^+^ epicardial cell layer was present in the normal heart and the sham-treated ICM hearts (Sham group), while these epicardial cells disappeared by day 1 after FG-aided epicardial placement of MSCs (Fig. 2c). In response to this finding, we found vascular formation in the FG-MSC complex, which included host-derived (DiI-negative) PECAM1^+^ endothelial cells (Fig. 2d). These data suggest that the epicardium would not block cellular and molecular interactions between the FG-MSC complex and the myocardium, including the MSC-derived paracrine mechanism. In addition, there were PECAM1/DiI double positive cells within the FG-MSC complex, suggesting differentiation of donor MSCs to endothelial cells forming a new vascular network.

### FG-aided epicardial placement of MSCs upregulated reparative genes more extensively

**Figure 3:**
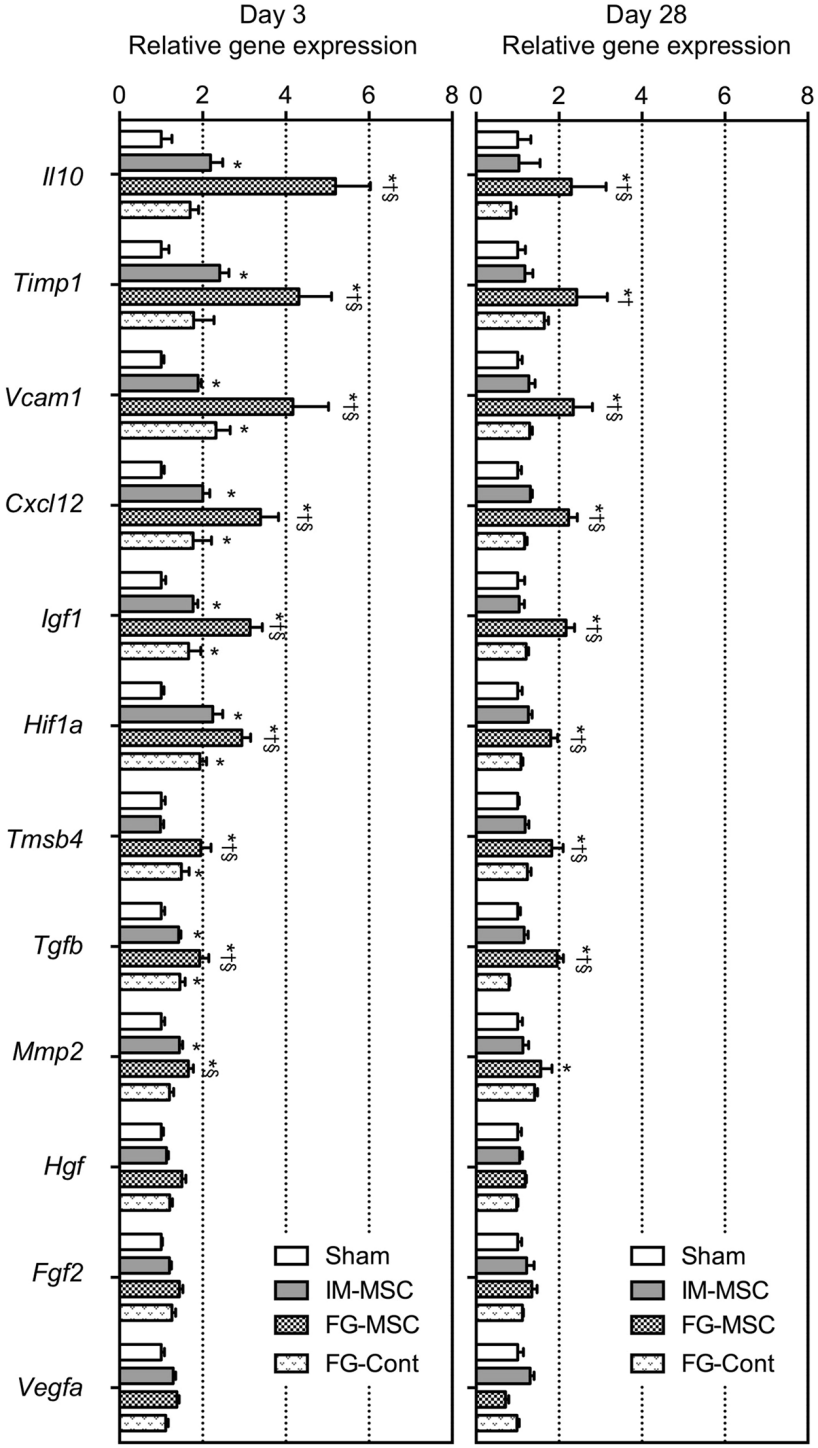
Enhanced upregulation of reparative genes after FG-aided, epicardial placement of MSCs. Real time RT-PCR analysis detected increased expression of multiple reparative genes in the FG-MSC group, as compared to the Sham (ICM with no treatment), IM-MSC, and FG-Cont (epicardial placement of FG without MSCs) groups at day 3 (left panel) and day 28 (right panel). Relative expression to the Sham group is shown. *p < 0.05 *vs*. the Sham group, ^†^ *p* < 0.05 *vs*. IM-MSC group. ^§^ *p < 0*.*05 vs*. FG-Cont group. n = 6 hearts in each group.

We then investigated whether FG-aided, instant epicardial placement indeed increased upregulation of reparative factors relevant to myocardial repair. Quantitative RT-PCR analysis demonstrated that myocardial expression of a range of reparative factors, including *Il10*, *Timp1*, *Vcam1*, *Cxcl12*, *Igf1*, *Hif1a* and *Tgfb*, was upregulated in the IM-MSC group at day 3 compared with the Sham group (Fig. 3). This upregulation was more evident in the FG-MSC group at day 3. Furthermore, the majority of these genes remained upregulated by day 28 in the FG-MSC group, while those in the IM-MSC group returned to the baseline values equivalent to the Sham group. The FG-Cont group (epicardial placement of FG only in the ICM rat) induced upregulation of a subset of these genes, including *Vcam1*, *Cxcl12*, *Igf1*, *Hif1a*, *Tmsb4*, and *Tgfb* compared to the Sham group. However, this upregulation was much less extensive and not sustained compared to that of the FG-MSC group.

### FG-aided epicardial placement of MSCs augmented myocardial tissue repair

**Figure 4:**
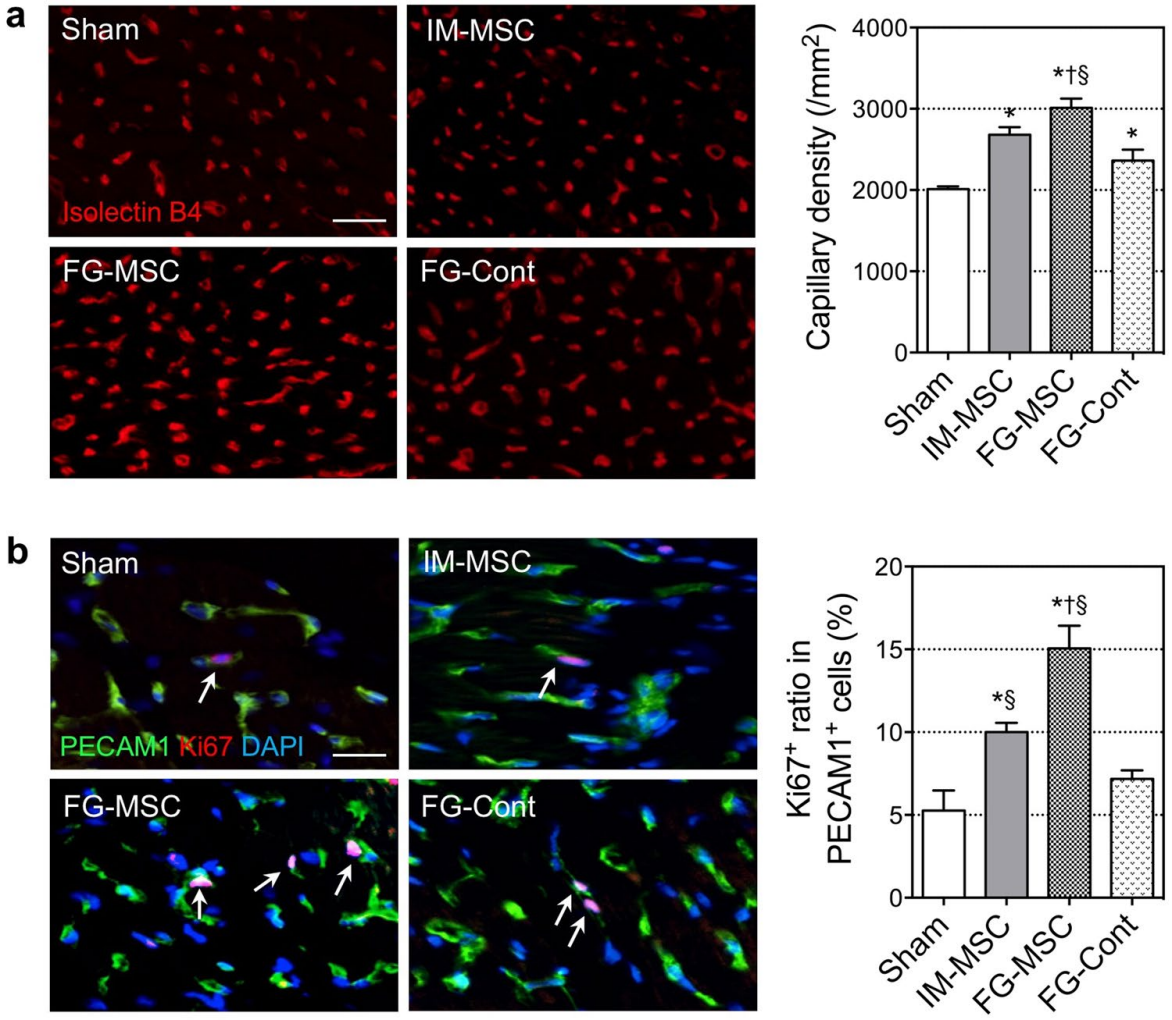
Enhanced microvasculature formation after FG-aided, epicardial placement of MSCs. (**a**) Isolectin B4 staining detected an increased capillary density in the peri-infarct viable area in the FG-MSC group, as compared to the Sham, IM-MSC and FG-Cont groups at day 7. Scale bar = 50 μm. (**b**) Co-immunohistostaining for PECAM1 and Ki67 detected an increased percentage of PECAM1^+^Ki67^+^ proliferating endothelial cells in the peri-infarct area of the FG-MSC group compared to the Sham, IM-MSC and FG-Cont groups at day 7. White arrows indicate PECAM1^+^Ki67^+^ cells. Scale bar = 20 μm. n = 6 hearts in each group. *p < 0.05 *vs*. Sham group, ^†^ *p* < 0.05 *vs*. IM-MSC group. ^§^ *p < 0*.*05 vs*. FG-Cont group.

**Figure 5:**
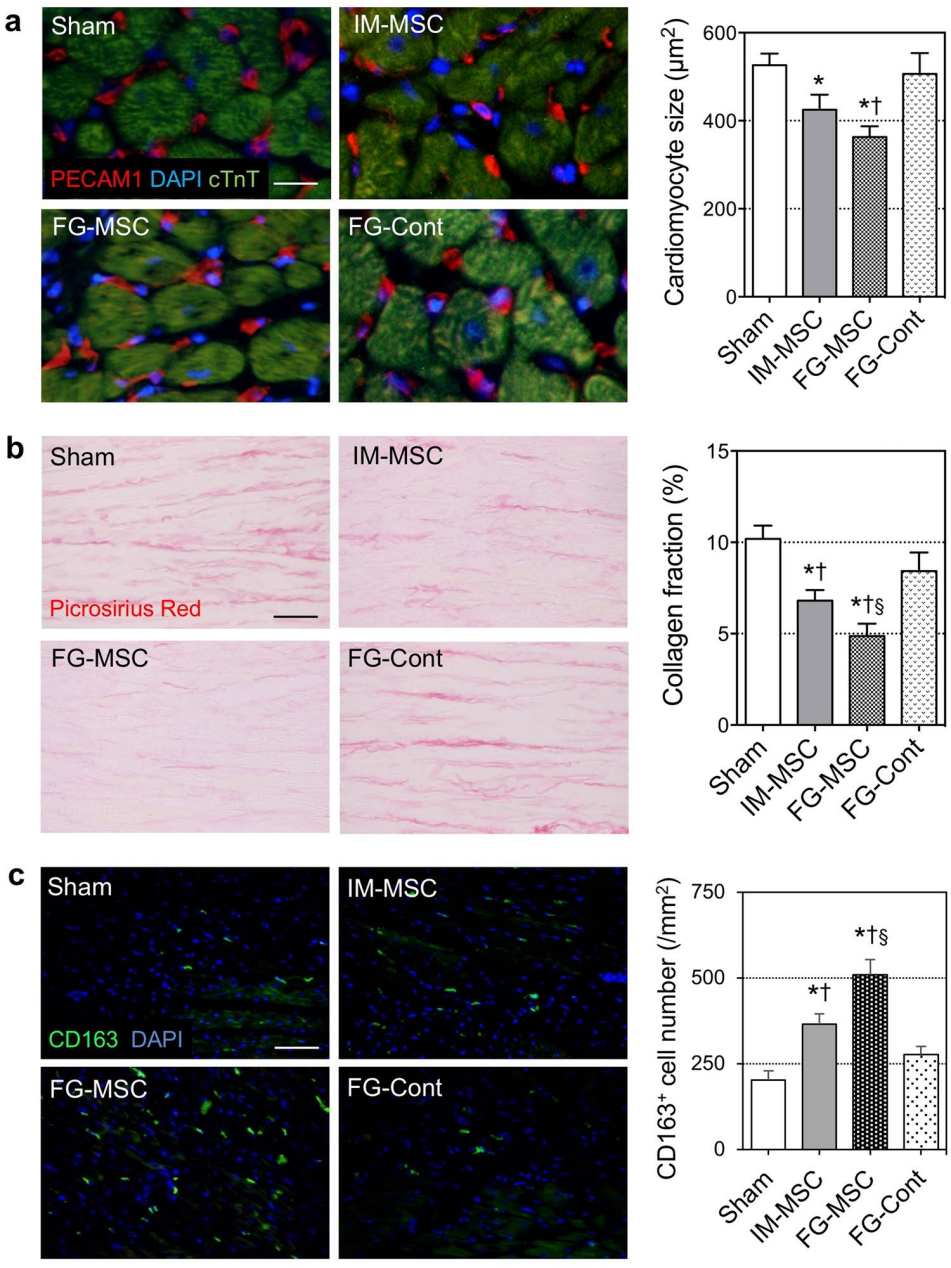
Attenuated adverse ventricular remodeling after FG-aided, epicardial placement of MSCs in the ICM rat heart. (**a**) Cross-sectional area of cTnT cardiomyocyte was measured using immunohistolabeling samples at day 28 post-treatment. Scale bar = 50 μm. (**b**) Post-MI interstitial fibrosis in the peri-infarct area was assessed by picrosirius red staining at day 28 post-treatment. Scale bar = 100 μm. (**c**) Accumulation of CD163^+^ cells (green) in the myocardium at day 7 was most evident in the FG-MSC group. Scale bar = 100 μm. n = 6 hearts in each group. *p < 0.05 *vs*. the Sham group, ^†^ *p* < 0.05 *vs*. IM-MSC group. ^§^ *p < 0*.*05 vs*. FG-Cont group.

Corresponding to the upregulation of reparative genes as described above, histological analysis identified enhancement of tissue repair of the failing myocardium in the ICM rat hearts after FG-aided, instant epicardial placement of MSCs. At day 28 after treatment, Isolectin B4 staining indicated an increased capillary density in the peri-infarct area in the FG-MSC group compared to all other groups (Fig. 4a). In support of this observation, the percebtage of Ki67^+^PECAM1^+^ proliferating endothelial cells was increased in the FG-MSC group compared to all other groups (Fig. 4b). Post-MI cardiomyocyte hypertrophy and interstitial collagen deposition in the peri-infarct areas were both attenuated in the FG-MSC group, compared to all other groups (Fig. 5a,b). Recently, it was suggested that alternative activated macrophages play an important role in the MSC-mediated paracrine effects as a secondary effector^19^. Consistent with this, we observed an accumulation of CD163^+^ alternatively activated macrophages in the peri-infarct area most notably in the FG-MSC group (Fig. 5c). The IM-MSC group also exhibited a reduction in cardiomyocyte hypertrophy, decrease in collagen deposition, and increase in CD163^+^ cell accumulation compared to the Sham group. However, the degrees of these effects in the IM-MSC group were less extensive when compared to the FG-MSC group.

### FG-aided epicardial placement of MSCs further enhanced cardiac function

Myocardial repair by FG-aided, instant placement of MSCs resulted in improved cardiac function recovery in the rat ICM model, compared to IM injection of MSCs (Table 1). At day 28 after MSC transplantation, echocardiography demonstrated that left ventricular ejection fraction (LVEF) and left ventricular fractional shortening (LVFS) in the FG-MSC and IM-MSC groups were increased as compared to the Sham group, with a greater increase measured for the FG-MSC group. In addition, the FG-MSC group showed reduced LV diameters compared to the Sham group. There was no significant difference in LVEF, LVFS, or LV dimensions between the Sham and FG-Cont groups. Heart rates were comparable among groups for echocardiography and catheterization. Baseline values prior to MSC transplantation were equivalent between groups. Consistently, cardiac catheterization showed the highest developed pressure, max dP/dt and min dP/dt, in the FG-MSC group among the group studied (Table 1). Left ventricular end-diastolic pressure was also reduced in the FG-MSC group, compared to other groups.

**Table 1:** Pre- and post-treatment cardiac function assessed by echocardiography and cardiac catheterization.

Echocardiography pre-treatment (=4 weeks after MI)						
Group	n	HR (bpm)	LVEF (%)	LVFS (%)	LVDs (mm)	LVDd (mm)
Sham	12	390.5 ± 8.7	36.2 ± 1.3	18.2 ± 0.7	6.9 ± 0.2	8.3 ± 0.1
IM-MSC	13	399.0 ± 6.7	35.8 ± 1.4	18.0 ± 0.8	7.0 ± 0.1	8.5 ± 0.1
FG-MSC	13	392.6 ± 6.8	35.8 ± 1.0	18.2 ± 0.5	6.9 ± 0.2	8.4 ± 0.2
FG-Cont	12	383.6 ± 7.9	35.4 ± 1.2	17.7 ± 0.7	6.7 ± 0.1	8.4 ± 0.1
**Echocardiography at 4 weeks after MSC therapy (=8 weeks after MI)**						
Group	n	HR (bpm)	LVEF (%)	LVFS (%)	LVDs (mm)	LVDd (mm)
Sham	12	394.7 ± 8.3	33.3 ± 1.5	16.3 ± 0.8	7.7 ± 0.2	9.2 ± 0.2
IM-MSC	13	393.1 ± 6.6	38.9 ± 1.7*^†^	20.2 ± 1.0*^†^	7.1 ± 0.2*^†^	8.9 ± 0.2
FG-MSC	13	399.6 ± 6.2	43.0 ± 1.4*^†§^	23.2 ± 0.8*^†§^	6.6 ± 0.1*^†§^	8.6 ± 0.2*
FG-Cont	12	387.6 ± 8.3	34.2 ± 1.6	16.7 ± 1.0	7.5 ± 0.2	9.0 ± 0.2
**Cardiac catheterization at 4 weeks after MSC therapy (=8 weeks after MI**)						
Group	n	HR (bpm)	Dev Pressure (mmHg)	LVEDP (mmHg)	Max dP/dt (mmHg/s)	Min dP/dt (mmHg/s)
Sham	12	380.5 ± 2.6	106.2 ± 3.8	10.2 ± 2.0	6,463 ± 265	−6,484 ± 234
IM-MSC	13	391.7 ± 4.2	120.1 ± 4.0*	8.7 ± 1.2	7,735 ± 275*^†^	−6,950 ± 198*
FG-MSC	13	388.7 ± 5.9	138.5 ± 3.9*^†§^	6.3 ± 0.8*^†§^	8,044 ± 268*^†§^	−7,705 ± 262*^†§^
FG-Cont	12	379.8 ± 4.0	111.9 ± 3.7	9.2 ± 1.3	6,876 ± 321	−6,773 ± 314

*Sham group*; Sham surgery (open chest only) at 4 weeks after MI

*IM-MSC group*; intramyocardial MSC injection at 4 weeks after MI

*FG-MSC group*; epicardial placement of FG incorporating MSC at 4 weeks after MI

*FG-Cont group*; epicardial placement of FG without MSC at 4 weeks after MI

*HR*, heart rate; *bpm*, beat per minute; *LVEF*, left ventricular ejection fraction; *LVFS*, left ventricular fractional shortening; *LVDs*, left ventricular end-systolic dimension; *LVDd*, left ventricular end-diastolic dimension; *Dev Pressure*, left ventricular developed pressure; *LVEDP*, left ventricular end-diastolic pressure.

*p < 0.05 *vs*. Sham group, ^†^ *p* < 0.05 *vs* ^.^ FG-Cont group, ^§^ *p* < 0.05 *vs*. IM-MSC group, Mean ± SEM.

## Discussion

This study demonstrated feasibility and therapeutic efficacy of FG-aided, instant epicardial placement of MSCs in a rat model of ICM. It was feasible and reproducible to instantly produce the FG incorporating MSC complex directly on the heart surface. This mixture promptly underwent gelation to form the FG-MSC complex, which firmly adhered to the targeted epicardial surface, enabling enhanced initial retention of MSCs compared to IM injection. No suture, additional glue or pericardial patch was required to fix the complex onto the heart, unlike epicardial placement of prefabricated FG constructs^15,16^. In addition, subsequent loss of retained MSCs was attenuated after FG-aided epicardial placement. These increased initial retention and improved maintenance of retained MSCs together amplified the quantity of engrafted MSCs, which corresponded to augmented myocardial tissue repair and improved cardiac function recovery. This enhanced therapeutic effect correlated with amplified myocardial upregulation of tissue repair-related genes. Freshly mixed suspension composed of fibrinogen, thrombin and MSCs is malleable enough to tightly fill the uneven heart surface. Such close-fitting adhesion would be beneficial for allowing permeation of biological factors secreted from donor MSCs into the myocardium. Our histological studies detected that the epicardial layer, which might block communications between MSCs and the myocardium, disappeared shortly after epicardial placement of the FG-MSC complex. This would allow permeation of the donor MSC secretome into the damaged myocardium. Collectively, FG-aided, on-site, instant epicardial placement of MSCs augmented initial retention and subsequent engraftment of MSCs, offering an enhanced therapeutic efficacy for ICM.

The enhanced donor cell quantity by FG-based cell transplantation has been reported previously^13,14^, and our results uncovered that this was a combined result from both increased initial retention and reduced subsequent loss of donor MSCs. Increased initial retention was achieved mainly by the unique nature of fibrin, which allowed the FG-MSC complex to firmly adhere to the heart, while securely retaining MSCs within. This feature attenuated the initial leakage of donor cells after transplantation, which is a major cause of the poor donor cell engraftment following IM and intracoronary injection^10,20^. Our *in vivo* results revealed that almost all transplanted MSCs were retained at 1 hour after FG-aided epicardial placement, while in contrast less than a half of donor cells were retained after IM injection of the MSC suspension. In addition, we uncovered attenuation of subsequent loss of donor MSCs both between day 3 and 7 and between day 7 and 28 after FG-aided epicardial placement compared to IM injection, additionally contributing to the increased quantity of engrafted donor MSCs. Underpinning this effect, apoptosis of transplanted MSCs was reduced after FG-aided epicardial placement compared to IM injection, while proliferation of transplanted MSCs rarely occurred in either cell-delivery method. Also, it is unlikely that engrafted, surviving MSCs are flushed out or migrate out from the heart at these later phases post-transplantation^20^. Collectively, the attenuation of subsequent loss of donor MSCs by FG-aided epicardial placement can be explained by reduced donor cell death. We speculate that increased survival of donor MSCs may be achieved by multiple FG-mediated mechanisms. Firstly, FG is known to provide a suitable three-dimensional scaffold to MSCs^21,22^, maintaining the high viability of incorporated MSCs. Secondly, we could expect FG to protect incorporated MSCs from external hazards, i.e. free radicals, by acting as a physical barrier. Thirdly, as reported in previous papers^22,23^, fibrin-based culture amplifies expression of anti-apoptotic factors in MSCs, including HIF1α and HGF, which would contribute to protection from insult in an autocrine manner^24^. Finally, while IM injection is known to induce myocardial inflammation that results in donor cell death^7,8^, epicardial placement does not cause such harmful inflammation.

Another possible mechanism by which FG-aided epicardial placement of MSCs achieved the augmented therapeutic effect might be enhancement of donor cell function *via* interactions with FG. As stated above, it has been reported that culture with fibrin increases the secretion of reparative factors, at least including HIF1α and HGF, from MSCs^22,23^. These upregulated factors could also play a role in the MSC-derived paracrine effect for myocardial repair^25–27^. Although the molecular mechanism underpinning the interaction between FG and MSCs that leads to the upregulation of reparative factors in MSCs is not certain, this may be explained by a characteristic component of the fibrin matrix, arginine-glycine-asparagine (RGD). RGD domain is known to bind integrin families expressed on the cell membrane of MSCs to mediate their intracellular signal cascades, resulting in amplified expression of reparative genes^28–31^. Further focused studies are needed to clarify the mechanism of FG-mediated enhancement of donor MSC function for myocardial repair.

Considering the clinical translation, FG-aided, instant epicardial placement of MSCs may be convenient and effective if added to coronary artery bypass grafting (CABG). The exposed heart during CABG offers an ideal opportunity to perform this MSC therapy in the safest and most reproducible manner. Although CABG is routinely performed to treat ICM patients, its outcome is often not satisfactory^32^. Of note, an addition of adult stem cell therapy is proven to augment the efficacy of CABG^33,34^. In the clinical scenario, an FG kit (containing fibrinogen and thrombin solutions) and ready-to-use allogeneic MSCs may be delivered from the manufacturer and/or hub stem cell center, obviating the necessity of a clinical-grade, cell processing facility at each hospital where the treatment is undertaken. At the end of bypass anastomosis, FG-aided, instant epicardial placement of MSCs can be easily completed in a short time. Thus, this highly accessible and effective treatment (CABG + FG-aided, instant epicardial MSC placement) has a great potential to be a widely-adopted standard treatment for ICM. In addition, other applications of FG-aided, instant epicardial MSC placement, including sole application as well as combination with other heart surgery, i.e. left ventricular assist device implantation and valve repair/replacement, for the treatment of ischemic and non-ischemic heart failure, are also promising.

One possible limitation in our study may be the use of the fluorescent dye (CM-DiI) for histological donor cell trafficking. Although utility of this labeling method for over 4 weeks has been repeatedly reported^2–4,7,8^, we cannot exclude the possibility that CM-DiI might be released from dead/damaged donor MSCs and be subsequently taken up by host cells including macrophages. As such, to provide a reliable and quantitative assessment of donor cell retention/survival, we have carefully performed qPCR for *Sry*. Actually, the results of donor cell presence were consistent between the CM-DiI-based histological and PCR assessments. Other concerns associated with epicardial placement of the FG-MSC complex may include a risk of altered hemostasis, cardiac tamponade or adverse ventricular contractile effect. However, we did not observe bleeding, clot formation or pericardial fluid collection in either FG-MSC or FG-Cont groups at 1 hour, 3 days or 28 days. Furthermore, we have confirmed that LVEDP was the lowest, and min dP/dt was the most improved in the FG-MSC group among all groups studied at Day 28. This indicates that epicardial placement of the FG-MSC complex will not cause significant adverse constrictive force to the heart. Given our result showing that fibrin glues were dissolved and absorbed by day 28 after placement, with loss of the majority of donor cells (Fig. 1c), it is unlikely that any adverse constrictive effect would occur after this period. Finally, it will be interesting to investigate whether the effect of FG to improve therapeutic effects of cell therapy is limited to bone marrow-derived MSCs or widely applicable to other donor cell types, i.e. adipose tissue-derived MSCs and cardiac resident progenitor cells.

In summary, the results of this study represent the proof-of-concept data indicating the feasibility and efficacy of FG-aided, instant epicardial placement of MSCs for the treatment of ICM. The use of FG offers multiple benefits to this approach, including not only improvement of initial retention but also maintenance of retained cells. This collectively results in enhanced therapeutic effects of this advanced MSC-based therapy compared to the current cell-delivery method. Further pre-clinical and clinical development of this user-friendly, highly-effective MSC-based therapy for ICM is warranted.

## Materials and Methods

All studies were performed with the approval of the institutional ethics committee at Queen Mary University of London and the Home Office, UK. The investigation conforms to the Principles of Laboratory Animal Care formulated by the National Society for Medical Research and the Guide for the Care and Use of Laboratory Animals (US National Institutes of Health Publication, 1996). Assessments were carried out in a blinded manner wherever possible. Sample numbers are stated in Figure Legends or Table.

### Isolation, culture and labeling of rat bone marrow-derived MSCs

MSCs were isolated from the bone marrow of the tibias and femurs of male Lewis rats (100–150 g, Charles River UK, Margate, UK) and expanded as described previously^2–4,35^. Isolated cells were cultured in αMEM with 20% inactivated fetal bovine serum containing L-glutamine, penicillin and streptomycin, at 37 °C in a humidified atmosphere containing 95% air and 5% CO_2_. (incubator: Binder, Germany). Culture medium was aspirated and replaced every 2–3 hours without additional washing. When cell confluency reached 80–90%, cells were passaged by detachment using 0.25% Trypsin in 0.2% EDTA (Sigma). Plating concentrations for subsequent passages were approximately 1 × 10^4^ cells/cm^2^. MSCs at passage 3 or 4 were used for all studies.

For tracking of donor cells, MSCs were labeled with a fluorescent dye, CM-DiI (Molecular Probes, Paisley, UK) as previously described^2–4^. We confirmed that >95% of the cells were positively stained with <2% cell death prior to transplantation (data not shown).

### Characterization of rat bone marrow-derived MSCs

For cell-surface marker characterization using flow cytometry, 1 × 10^6^ MSCs were stained with FITC-conjugated anti-CD34 (Santa Cruze, USA), CD45 (Chemicon; Hampshire, UK), CD90 (Abcam, Cambridge, UK) or Alexa 647-conjugated anti-CD29 (Biolegend, London, UK) antibodies. Corresponding isotype-matched control antibodies were used for negative controls. All antibodies were used at 1:100 dilution following manufacturer’s instructions. Samples were analyzed using the Dako Cyan flow-cytometer (Dako Cytomation, UK).

In addition, to confirm their osteogenic and adipogenic differentiation potential, MSCs were plated in 24-well plates and subjected to adipogenic or osteogenic differentiation conditions^2,3^. Adipogenic differentiation medium was α-minimal essential medium (α-MEM) supplemented with 100 μM isobutyl methylxanthine (Sigma-Aldrich, UK), 60 μM indomethacin (Fluka; Dorset, UK), 1 μg/ml insulin (Sigma-Aldrich), and 0.5 μM hydrocortisone (Sigma-Aldrich), while osteogenic differentiation medium was α-MEM supplemented with 0.1 μM dexamethasone (Sigma-Aldrich), 10 mM β-glycerophosphate (Sigma-Aldrich), and 0.05 mM ascorbic acid (Sigma-Aldrich). Medium was changed every 2–3 days. After 3 weeks of culture, cells were fixed with 4% paraformaldehyde, and stained with Oil red O (Fluka) for detection of adipocytes containing lipid vacuoles or with Alizarin red (Fluka) to detect osteocytes containing calcium deposits.

As a result, MSC-specific surface marker expression (CD90^+^/CD29^+^/CD45^−^/CD34^−^) and differentiation abilities to adipocytes and osteocytes were detected, confirming MSC identity (Supplemental Fig. S2).

### Induction of ICM rat model and MSC transplantation

MI was induced in female Lewis rats (150–200 g; Charles River UK) by ligating the left coronary artery through left thoracotomy under isoflurane anesthesia and mechanical ventilation as described previously^3,4,8^. At 4 weeks, rats were subjected to echocardiography, and those showing inappropriate left ventricular ejection fraction (LVEF > 45% or < 25%) were excluded from the study^3,4,8^. Included animals were randomly allocated into 4 treatment groups. For the FG-MSC group, the heart was exposed through re-left thoracotomy under mechanical ventilation. Then, 40 μl of phosphate-buffered saline (PBS) containing 174 mg/ml human fibrinogen, 60 U/ml human coagulation factor XIII, and 1000 KIU/ml bovine aprotinin, in which 4 × 10^6^ MSCs from male Lewis rat bone marrow were suspended, was added onto the epicardial heart surface, together with 40 μl of 5.88 mg/ml calcium chloride solution containing 500 IU/ml human thrombin, in a dropwise manner using two micropipettes, aiming to cover the infarct and surrounding peri-infarct myocardial areas. These solutions were from a Beriplast P Combi-set (CSL Behring). The volume of each solution was chosen as a result of a pilot study. For the IM-MSC group, 4 × 10^6^ of MSCs were suspended in 200 μl PBS and intramyocardially injected into two sites (100 μl each), targeting the border and infract areas as we have previously described^2^, The Sham group received an open/close chest procedure at 4 weeks after coronary artery ligation, whilst the FG-Cont group consisted of FG (no MSC) placed on the heart surface to cover the infarct and surrounding peri-infarct areas in the same manner as in the FG-MSC group.

### Cardiac function measurement

Transthoracic echocardiography was performed at 4 weeks after left coronary artery ligation (for pre-treatment data) and at day 28 post-treatment using Vevo-770 echocardiography machine (VisualSonics, Amsterdam, Netherlands) under 1.5% isoflurane inhalation via a nose cone^3,4^. LVEF and LVFS were calculated from the data obtained with 2-dimensional tracing. All data were collected from at least 3–5 different measurements in a blinded manner. In addition, hemodynamic parameters were measured by using cardiac catheterization (SPR-320 and PVAN3.2; Millar Instruments, Houston, TX) as previously described^3,4^. Briefly, under general anesthesia using 1.5% isoflurane inhalation and mechanical ventilation, the catheter was inserted into the left ventricular cavity through the right common carotid artery. Intra-LV and intra-aortic pressure signals were measured with a transducer and conditioner (MPVS-300; Millar Instruments) and digitally recorded with a data acquisition system (PowerLab 8/30; ADInstruments, Oxford, UK). All data were collected from at least 5 different measurements per animal in a blinded manner.

### Quantitative assessment of donor cell presence

To quantify the presence of engrafted male rat MSCs in the syngeneic female rat heart, presence of the Y chromosome–specific *Sry* gene was quantitatively assessed by TaqMan real-time PCR (Prism 7900HT; Applied Biosystems)^2–4,7^. At the chosen time after treatment, the left ventricular myocardium was collected (with removal of atriums and right ventricular free walls), genomic DNA extracted using the DNeasy Blood&Tissue kit (Qiagen), and *Sry* analysis performed in technical duplicate. The signal in each sample was normalized to the amount of DNA by measuring the autosomal single-copy gene *Spp1* (*osteopontin)* as an internal standard. To generate a standard curve, left ventricular myocardium from a female rat at day 28 after left coronary artery ligation were mixed with either 1 × 10^7^, 1 × 10^6^, 1 × 10^5^ or 1 × 10^4^ of male rat MSCs, and processed for *Sry* analysis (n = 3). Donor cell loss between day A and B was calculated using obtained donor cell presence as follows:

% loss of donor cells between day A and day B = (donor cell presence at day A − donor cell presence at day B) ÷ donor cell death at day A × 100.

### Histological analysis

The hearts were harvested, fixed with 4% paraformaldehyde, and frozen in OCT compound using liquid nitrogen. Cryosections were cut and incubated with polyclonal anti-cardiac troponin-T antibody (1:200 dilution; HyTest, Turku, Finland), polyclonal anti-cleaved caspase-3 antibody (1:250, Cell Signaling), biotin-conjugated Griffonia simplicifolia lectin I-isolectin B4 (1:100; Vector Laboratories, Peterborough, UK), monoclonal anti-PECAM1 antibody (1:50; AbD Serotec, Kidlington, UK), monoclonal anti-ICAM1 antibody (1:50, Abcam), monoclonal anti-Ki67 antibody (1:50, Dako), or monoclonal anti-CD163 antigen antibody (1:200; AbD Serotec) at 4 °C overnight. This was followed by visualization using fluorophore-conjugated secondary antibodies (Life Technologies) for 1 hour at room temperature. Samples were analyzed by fluorescence microscopy (BZ8000; Keyence, Milton Keynes, UK) with or without nuclear counterstaining using 4′,6-diamidino-2-phenylindole (DAPI). For semi-quantitative assessments, ten fields (either peri-infarct or remote areas in the interventricular septum) per heart were randomly selected and assessed. To evaluate cardiomyocyte size, the cross-sectional area of appropriately detected cTnT^+^ cardiomyocytes (transversely cut; having central nuclei and surrounded by circle-shaped capillaries) was measured for 50 cardiomyocytes per area.

Another set of sections were stained with 0.1% picrosirius red (Sigma-Aldrich) to semi-quantify extra-cellular collagen deposition using NIH imageJ-analysis software^2,7,36^. In addition, staining with Oil red O (Sigma-Aldrich) and Alizarin red (Sigma-Aldrich) was performed for detecting adipogenic and osteogenic differentiation, as previously described^2–4^.

### Analysis of gene expression

Total RNA was extracted from left ventricular walls (free walls and interventricular septum) using the RNeasy Mini kit (Qiagen) with DNase I treatment, and converted to cDNA using the High Capacity cDNA Reverse Transcription kit (Applied Biosystem) according to the manufacturer’s instruction. Expression of chosen genes relevant to myocardial repair/regeneration was assessed by quantitative RT-PCR (Prism 7900HT, Applied Biosystems) as previously described^2–4,7^. All TaqMan primers and probes were purchased from Applied Biosystems with an exception of *Tmsb4* (Sigma-Aldrich). Expression was normalized to *Ubc*.

### Statistical analysis

All values are expressed as mean ± SEM. Statistical comparison of multiple groups were performed with one-way ANOVA followed by the Least Significant Difference test, except for Fig. 1a with repeated measures ANOVA. Comparison of two groups was performed using the student’s unpaired *t*-test. A value of *p* < 0.05 was considered statistically significant.

## Electronic supplementary material

Supplementary figures

## References

[1] Narita, T. & Suzuki, K. Bone marrow-derived mesenchymal stem cells for the treatment of heart failure. *Heart Fail. Rev.* **20**, 53–68 (2015). 24862087 10.1007/s10741-014-9435-x

[2] Narita, T. *et al*. The use of scaffold-free cell sheet technique to refine mesenchymal stromal cell-based therapy for heart failure. *Mol. Ther.* **21**, 860–867 (2013). 23358187 10.1038/mt.2013.9 PMC3616531

[3] Tano, N. *et al*. Epicardial placement of mesenchymal stromal cell-sheets for the treatment of ischemic cardiomyopathy; *in vivo* proof-of-concept study. *Mol. Ther.* **22**, 1864–1871 (2014). 24930600 10.1038/mt.2014.110 PMC4428395

[4] Tano, N. *et al*. Allogeneic Mesenchymal Stromal Cells Transplanted Onto the Heart Surface Achieve Therapeutic Myocardial Repair Despite Immunologic Responses in Rats. *J*. *Am*. *Heart Assoc*. **5**, 10.1161/JAHA.115.002815 (2016). 10.1161/JAHA.115.002815 PMC4802488 26896478

[5] Langrzyk, A. *et al*. Critical View on Mesenchymal Stromal Cells inRegenerative Medicine. *Antioxid*. *Redox Signal*. Epub ahead of print (2017). 10.1089/ars.2017.7159 28874054

[6] Fukushima, S., Sawa, Y. & Suzuki, K. Choice of cell-delivery route for successful cell transplantation therapy for the heart. *Future Cardiol.* **9**, 215–227 (2013). 23463974 10.2217/fca.12.85

[7] Narita, T. *et al*. The use of cell-sheet technique eliminates arrhythmogenicity of skeletal myoblast-based therapy to the heart with enhanced therapeutic effects. *Int. J. Cardiol.* **168**, 261–269 (2013). 23046598 10.1016/j.ijcard.2012.09.081

[8] Fukushima, S. *et al*. Direct intramyocardial but not intracoronary injection of bone marrow cells induces ventricular arrhythmias in a rat chronic ischemic heart failure model. *Circulation* **115**, 2254–2261 (2007). 17438152 10.1161/CIRCULATIONAHA.106.662577

[9] Campbell, N. G. & Suzuki, K. Cell delivery routes for stem cell therapy to the heart: current and future approaches. *J. Cardiovasc. Transl. Res.* **5**, 713–726 (2012). 22648235 10.1007/s12265-012-9378-3

[10] Campbell, N. G. *et al*. Cell Size Critically Determines Initial Retention of Bone Marrow Mononuclear Cells in the Heart after Intracoronary Injection: Evidence from a Rat Model. *PLoS One* **11**, e0158232, 10.1371/journal.pone.0158232 (2016). 27380410 10.1371/journal.pone.0158232 PMC4933345

[11] Lee, M. G. & Jones, D. Applications of fibrin sealant in surgery. *Surg. Innov.* **12**, 203–213 (2005). 16224640 10.1177/155335060501200304

[12] Wu, X., Ren, J. & Li, J. Fibrin glue as the cell-delivery vehicle for mesenchymal stromal cells in regenerative medicine. *Cytotherapy* **14**, 555–562 (2012). 22175911 10.3109/14653249.2011.638914

[13] Christman, K. L. *et al*. Injectable fibrin scaffold improves cell transplant survival, reduces infarct expansion, and induces neovasculature formation in ischemic myocardium. *J. Am. Coll. Cardiol.* **44**, 654–660 (2004). 15358036 10.1016/j.jacc.2004.04.040

[14] Guo, H. D., Wang, H. J., Tan, Y. Z. & Wu, J. H. Transplantation of marrow-derived cardiac stem cells carried in fibrin improves cardiac function after myocardial infarction. *Tissue Eng. Part A* **17**, 45–58 (2011). 20673001 10.1089/ten.TEA.2010.0124

[15] Atluri, P. *et al*. Tissue-engineered, hydrogel-based endothelial progenitor cell therapy robustly revascularizes ischemic myocardium and preserves ventricular function. *J. Thorac. Cardiovasc. Surg.* **148**, 1090–1097 (2014). 25129603 10.1016/j.jtcvs.2014.06.038 PMC4155940

[16] Menasche, P. *et al*. Towards a clinical use of human embryonic stem cell-derived cardiac progenitors: a translational experience. *Eur. Heart J.* **36**, 743–750 (2015). 24835485 10.1093/eurheartj/ehu192

[17] Dib, N., Khawaja, H., Varner, S., McCarthy, M. & Campbell, A. Cell therapy for cardiovascular disease: a comparison of methods of delivery. *J. Cardiovasc. Transl. Res.* **4**, 177–181 (2011). 21181320 10.1007/s12265-010-9253-z PMC3047684

[18] Gnecchi, M., Zhang, Z., Ni, A. & Dzau, V. J. Paracrine mechanisms in adult stem cell signaling and therapy. *Circ. Res.* **103**, 1204–1219 (2008). 19028920 10.1161/CIRCRESAHA.108.176826 PMC2667788

[19] Ben-Mordechai, T. *et al*. Macrophage subpopulations are essential for infarct repair with and without stem cell therapy. *J. Am Coll. Cardiol.* **62**, 1890–1901 (2013). 23973704 10.1016/j.jacc.2013.07.057

[20] Suzuki, K. *et al*. Dynamics and mediators of acute graft attrition after myoblast transplantation to the heart. *FASEB J.* **18**, 1153–1155 (2004). 15155562 10.1096/fj.03-1308fje

[21] Perea-Gil, I., Prat-Vidal, C. & Bayes-Genis, A. *In vivo* experience with natural scaffolds for myocardial infarction: the times they are a-changin’. *Stem Cell Res. Ther.* **6**, 248, 10.1186/s13287-015-0237-4 (2015). 26670389 10.1186/s13287-015-0237-4 PMC4681026

[22] Kim, I. *et al*. Fibrin glue improves the therapeutic effect of MSCs by sustaining survival and paracrine function. *Tissue Eng. Part A* **19**, 2373–2381 (2013). 23701237 10.1089/ten.tea.2012.0665 PMC3807701

[23] Roura, S. *et al*. Human umbilical cord blood-derived mesenchymal stem cells promote vascular growth *in vivo*. *PLoS One* **7**, e49447, 10.1371/journal.pone.0049447 (2012). 23166670 10.1371/journal.pone.0049447 PMC3500294

[24] Lee, S., Choi, E., Cha, M. J. & Hwang, K. C. Cell adhesion and long-term survival of transplanted mesenchymal stem cells: a prerequisite for cell therapy. *Oxid. Med. Cell. Longev.* **2015**, 632902, 10.1155/2015/632902 (2015). 25722795 10.1155/2015/632902 PMC4333334

[25] Vandervelde, S., van Luyn, M. J., Tio, R. A. & Harmsen, M. C. Signaling factors in stem cell-mediated repair of infarcted myocardium. *J. Mol. Cell. Cardiol.* **39**, 363–376 (2005). 15992820 10.1016/j.yjmcc.2005.05.012

[26] Crisostomo, P. R. *et al*. Human mesenchymal stem cells stimulated by TNF-alpha, LPS, or hypoxia produce growth factors by an NF kappa B- but not JNK-dependent mechanism. *Am. J. Physiol. Cell Physiol.* **294**, C675–682 (2008). 18234850 10.1152/ajpcell.00437.2007

[27] Madrigal, M., Rao, K. S. & Riordan, N. H. A review of therapeutic effects of mesenchymal stem cell secretions and induction of secretory modification by different culture methods. *J. Transl. Med.* **12**, 260, 10.1186/s12967-014-0260-8 (2014). 25304688 10.1186/s12967-014-0260-8 PMC4197270

[28] Zhang, H., Lin, C. Y. & Hollister, S. J. The interaction between bone marrow stromal cells and RGD-modified three-dimensional porous polycaprolactone scaffolds. *Biomaterials* **30**, 4063–4069 (2009). 19487019 10.1016/j.biomaterials.2009.04.015 PMC4367542

[29] Yu, J. *et al*. The use of human mesenchymal stem cells encapsulated in RGD modified alginate microspheres in the repair of myocardial infarction in the rat. *Biomaterials* **31**, 7012–7020 (2010). 20566215 10.1016/j.biomaterials.2010.05.078

[30] Prowse, A. B., Chong, F., Gray, P. P. & Munro, T. P. Stem cell integrins: implications for *ex-vivo* culture and cellular therapies. *Stem Cell Res.* **6**, 1–12 (2011). 21075697 10.1016/j.scr.2010.09.005

[31] Chen, J., Crawford, R., Chen, C. & Xiao, Y. The key regulatory roles of the PI3K/Akt signaling pathway in the functionalities of mesenchymal stem cells and applications in tissue regeneration. *Tissue Eng. Part B Rev.* **19**, 516–528 (2013). 23651329 10.1089/ten.TEB.2012.0672

[32] Velazquez, E. J. *et al*. Coronary-artery bypass surgery in patients with left ventricular dysfunction. *N. Engl. J. Med.* **364**, 1607–1616 (2011). 21463150 10.1056/NEJMoa1100356 PMC3415273

[33] Donndorf, P. *et al*. Intramyocardial bone marrow stem cell transplantation during coronary artery bypass surgery: a meta-analysis. *J. Thorac. Cardiovasc. Surg.* **142**, 911–920 (2011). 21376346 10.1016/j.jtcvs.2010.12.013

[34] Karantalis, V. *et al*. Autologous mesenchymal stem cells produce concordant improvements in regional function, tissue perfusion, and fibrotic burden when administered to patients undergoing coronary artery bypass grafting: The Prospective Randomized Study of Mesenchymal Stem Cell Therapy in Patients Undergoing Cardiac Surgery (PROMETHEUS) trial. *Circ. Res.* **114**, 1302–1310 (2014). 24565698 10.1161/CIRCRESAHA.114.303180 PMC4104798

[35] Hayashi, Y. *et al*. Topical transplantation of mesenchymal stem cells accelerates gastric ulcer healing in rats. *Am. J. Physiol. Gastrointest. Liver Physiol.* **294**, G778–786 (2008). 18202110 10.1152/ajpgi.00468.2007

[36] Kaneko, M. *et al*. Extracellular high mobility group box 1 plays a role in the effect of bone marrow mononuclear cell transplantation for heart failure. *PLoS One* **8**, e76908, 10.1371/journal.pone.0076908 (2013). 24204700 10.1371/journal.pone.0076908 PMC3799896

