# Supplementary material for: Fibrin Glue-aided, Instant Epicardial Placement Enhances the Efficacy of Mesenchymal Stromal Cell-Based Therapy for Heart Failure
Source: Sci Rep. 2018 Jun 21;8:9448. doi: 10.1038/s41598-018-27881-5 (PMC6013428; doi:10.1038/s41598-018-27881-5)
Supplement: Supplementary file 1 — Supplementary figures [file 41598_2018_27881_MOESM1_ESM.pdf]

Supplementary figures

Fibrin Glue-aided, Instant Epicardial Placement Enhances the Efficacy of Mesenchymal Stromal Cell-Based Therapy for Heart Failure

Kazuya Kobayashi, Yuki Ichihara, Nobuko Tano, Laura Fields, Nilaani Murugesu, Tomoya Ito, Chiho Ikebe, Fiona Lewis, Kenta Yashiro, Yasunori Shintani, Rakesh Uppal, Ken Suzuki

William Harvey Research Institute, Barts and The London School of Medicine and Dentistry, Queen Mary University of London, London, United Kingdom

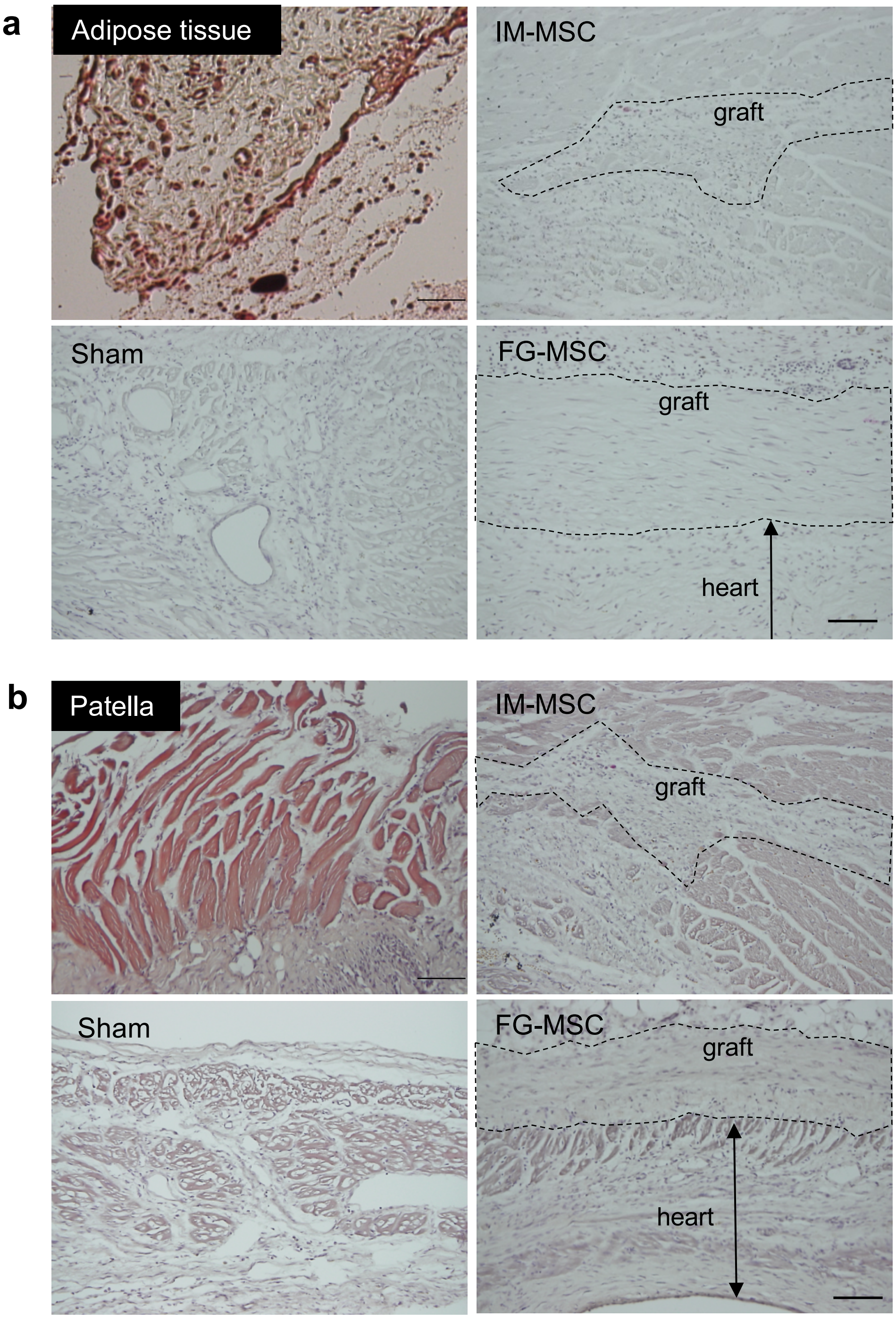

Supplemental Fig. S1. Engrafted MSCs did not undergo adipogenic or osteogenic differentiation in vivo.

(**a**) Oil red-O staining detected the existence of adipocytes (red~brown) in the adipose tissue (positive control); however, there was no Oil red-O-positive cells in the heart or MSC-grafts in any group studied. Dotted line areas show MSC-grafts. Representative images from 6 different hearts in each group are shown. Scale bars = 100 μm. Dotted line areas show MSC-grafts.

(**b**) Alizarin red staining showed calcium deposition (red) in the patella (positive control). No such signal was observed in the heart or donor MSC grafts in any group. Representative images from 6 different hearts in each group are shown. Scale bars = 100 μm.

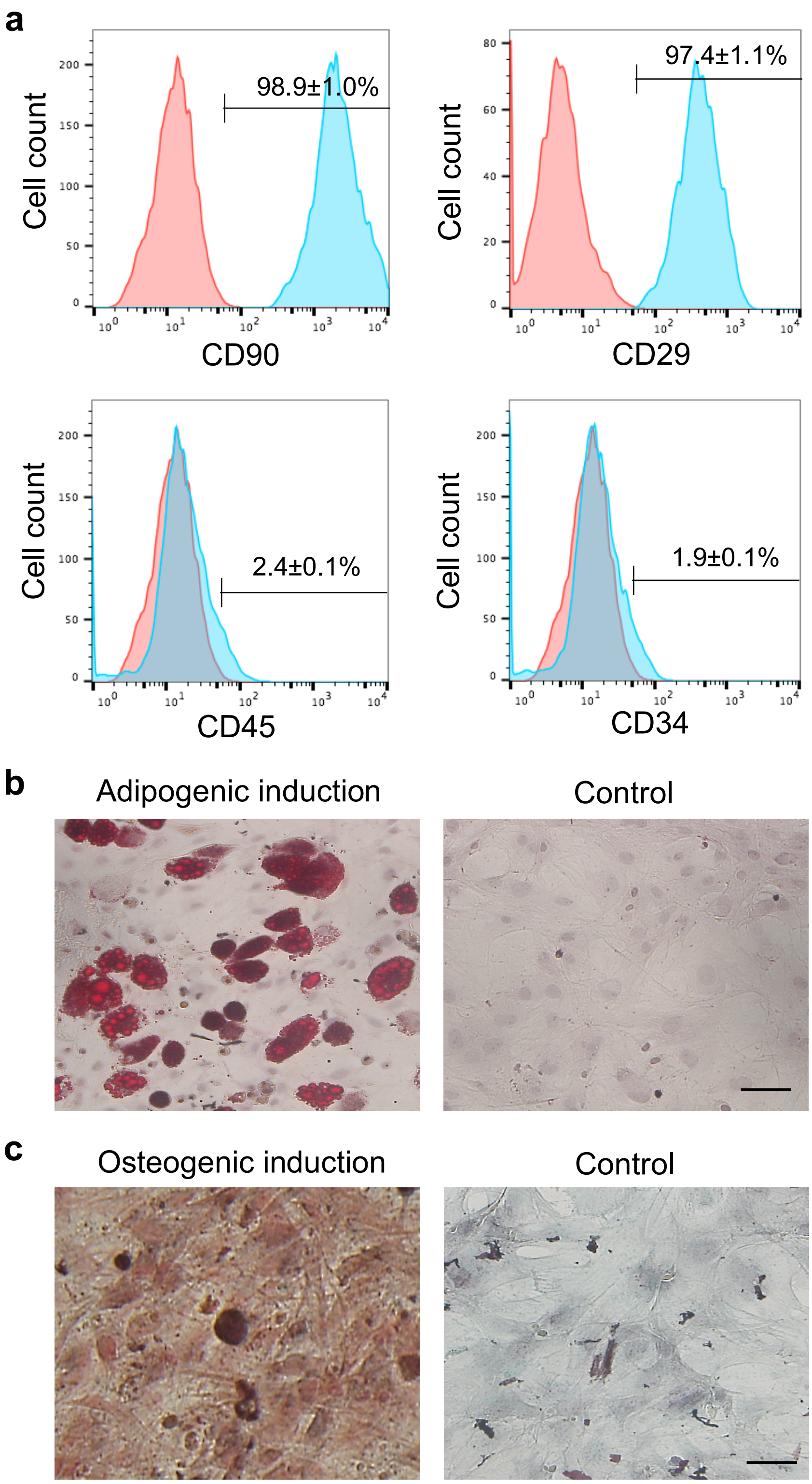

Supplemental Fig. S2. In vitro characterization confirmed the identity of rat bone marrow-derived MSCs.

(**a**) Flow cytometric analysis demonstrated that isolated cells from rat bone marrow expressed CD90 and CD29, but not CD45 or CD34. Red histograms indicate the control IgG data. Representative histograms from 4 different samples are presented.

(**b**) Oil red-O staining demonstrated that rat MSCs were able to differentiate to adipocytes under the adipogenic differentiation condition (left panel). Control (right panel) shows MSCs cultured in growth medium without adipogenic stimulation. Scale bar = 20 μm.

(**c**) Alizarin red staining showed that rat MSCs were able to differentiate to osteocytes under the osteogenic differentiation condition (left panel). Control (right panel) is a micrograph of MSCs cultured in growth medium without osteogenic stimulation. Scale bar = 20 μm.
